# Spontaneous subepithelial hemorrhage of renal pelvis and ureter (Antopol-Goldman lesion) in hemophilia A patient with inhibitor: Case report and review of the literature

**DOI:** 10.1097/MD.0000000000020851

**Published:** 2020-06-26

**Authors:** Taha Koray Sahin, Elifcan Aladag, Emil Setterzade, Gulay Sain Guven, Ibrahim Celalettin Haznedaroglu, Salih Aksu

**Affiliations:** a Department of Internal Medicine; b Department of Hematology; c Department of Radiology, Faculty of Medicine, Hacettepe University Ankara, Turkey.

**Keywords:** Antopol-Goldman lesion, hemophilia

## Abstract

**Introduction:**

The Antopol-Goldman lesion (AGL), which expresses subepithelial hemorrhage in the renal pelvis, was first defined by Antopol and Goldman in 1948. The objective of this study is to report the first case of AGL in patients with congenital hemophilia and review the relevant literature.

**Patient concerns:**

A 32-year-old male patient diagnosed with congenital hemophilia A (FVIII = %4) with high responding inhibitors (7.4 BU) was admitted to our emergency department with gross hematuria and sudden onset flank pain.

**Diagnosis:**

Abdominal computed tomography (CT-scan) presented a hyperdense lesion in the left ureteropelvic junction with Hounsfield Units of 56 compatibles with hemorrhage.

**Interventions:**

The patient was given 4500 IU of factor eight inhibitor bypass activity (FEIBA) intravenously twice daily for 5 days. Subsequently, 4500 IU of FEIBA was administrated once a day for 2 days.

**Outcomes:**

The patient's complaints disappeared on the fourth day of treatment. Macroscopic and microscopic hematuria was not seen in the following days. Follow-up CT was done 3 months after discharge and showed normal left renal pelvis without hyperdenosis. Follow-up CT was performed 3 months after discharge and presented normal left renal pelvis with no hyperdense lesion.

**Conclusion:**

Although very rare, AGL should be kept in mind in the differential diagnosis of renal pelvic hemorrhage. In the patient who has an underlying history of coagulopathy nephrectomy can be avoided when there is awareness of AGL.

## 1 Introduction

Antopol-Goldman Lesion (AGL), which states subepithelial hemorrhage in the renal pelvis, was first pronounced by Antopol and Goldman in 1948.^[1]^ The first cases are diagnosed after nephrectomy because they mimic renal neoplasm.^[2,3]^ Although this hemorrhage is extremely difficult to distinguish from the true renal pelvis tumors, the diagnosis can be established by radiologically and clinically. The patients may have with hematuria and flank pain. Conservative approach is preferred as a treatment in recent studies.^[4–6]^

Hemophilia A is an X-linked congenital bleeding disorder caused by factor VIII deficiency. Hematuria and renal-ureteral hemorrhage are the most common complications in hemophilia A patients.^[7]^ Prevention or treatment of bleeding is the replacement of VIII. However, inhibitor development is the most common and most serious complication in hemophilia A patients and it occurs in up to 30% of patients with severe hemophilia A.^[8]^ The current standard hemostatic agents for patients with inhibitor are recombinant activated factor VII (rFVIIa) and activated prothrombin complex concentrate (APCC).

To date, 39 cases have been reported due to severe factor V deficiency, overuse nonsteroidal anti-inflammatory drugs, trauma, and amyloidosis (Table 1).^[1–6,9–24]^ However, AGL had never been reported in any hemophilia patient. Therefore, the purpose of this study is to report the first case ever of AGL in a patient with congenital hemophilia and to review the relevant literature.

**Table 1:**
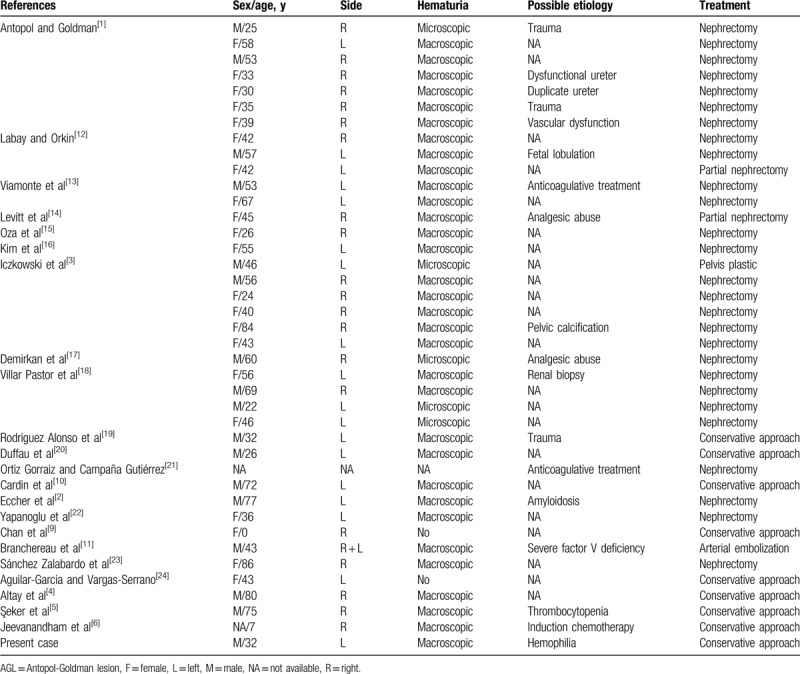
Summary of previously published cases of subepithelial pelvic hematoma (Antopol-Goldman lesion). Our present patient is the first case of hemophilia A with Antopol-Goldman lesion.

## 2 Case report

The patient has provided written informed consent for publication of the case.

**Figure 1:**
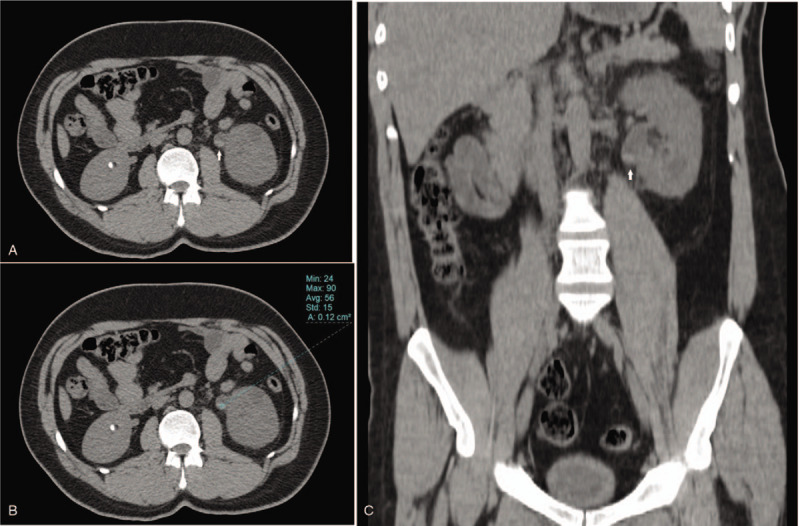
Axial (A, B) and coronal reformatted (C) unenhanced computed tomography (CT) images demonstrate hyperdense lesion in the left ureteropelvic junction (arrows). There is also small stone in the right kidney.

Thirty-two-year-old male patient with a history of severe hemophilia A was admitted presented to the emergency department with gross hematuria and sudden onset of side pain. His medical history was unremarkable except for hemophilia A. He was diagnosed at 1 month old and since then, he begins to administer factor VIII concentrates. However, 20 years after diagnosis, inhibitors were detected. Treatment with factor VIII concentrates is discontinued. Instead, rFVIIa and APCC are administered. During the disease, significant hemorrhages such as psoas hematoma and hemarthrosis were observed. He had no history of trauma or use of anticoagulant or anti-aggregant agents. The patient was transferred to the hematology service. Physical inspection-exposed tenderness was present on the left flank and lower abdominal quadrant, whereas other systemic evaluations were normal. In addition, his vital signs were within normal ranges. Preliminary laboratory results comprised a normal complete blood count, including platelets, prolonged activated partial thromboplastin time (aPTT), and prolonged prothrombin time (PT). FVIII had significantly reduced activity (4%; normal range, 60%–150%), and FVIII inhibitor had a high titer (7.4 Bethesda units [BU]/mL; normal range, 0–0.6 BU/mL). Abdominal computed tomography (CT) scan without intravenous contrast media shown a hyperdense lesion in the left ureteropelvic junction with Hounsfield Units of 56 compatibles with hemorrhage (Fig. 1).

**Figure 2:**
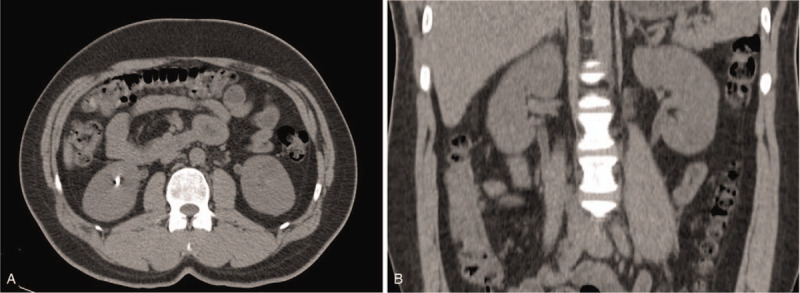
Computed tomography (CT) without intravenous contrast media shows complete resolution of the lesion at follow-up 3 months after discharge.

To control his bleeding, the patient was given 4500 IU of factor eight inhibitor bypass activity (FEIBA) intravenously twice daily for 5 days. He was given intravenous tramadol 100 mg/day for severe pain. The patient's complaints disappeared on the fourth day of treatment. The patient had no macroscopic and microscopic hematuria in the following days. Subsequently, 4500 IU FEIBA was administered once daily for 2 days and the patient was discharged from the hospital. Follow-up CT was done 3 months after discharge and showed normal left renal pelvis with no hyperdense lesion which confirmed the radiological diagnosis of AGL (Fig. 2). Although prophylactic treatment was not applied to the patient, no recurrence was observed at 1-year follow-up.

## 3 Discussion

AGL is a benign hemorrhagic lesion that is rarely seen with clinical appearance and imaging results that can mimic the tumor of the renal collection system. Although it can be seen in all age group, it is more common in the elderly. However, 1 neonatal case and 12 cases under 35 years of age have also been reported.^[9]^ The most common clinical appearance of suburothelial pelvic hemorrhage is macroscopic hematuria and acute side pain as in our patient.^[4]^ Although the etiopathogenesis is unclear in most of the previously reported cases, there are 39 cases of AGL in the literature due to various possible conditions related to the etiology of AGL, including trauma, congenital malformations (bifid pelvis, fetal lobulation, aberrant vessels, and ureteral insertion abnormalities), hypertension, excessive analgesic use, anticoagulant treatment, severe factor V deficiency, amyloidosis, thrombocytopenia, and induction chemotherapy (Table 1). None of these factors were present in our case. Sixteen patients were male, 21 were female and for 2 patients the sex was not reported. The median age was 43 years (0–86 years). Thirty-one cases presented with macroscopic hematuria (%81).

Hemophilia A is classified as severe (<1% factor activity), moderate (1-5% factor activity), and mild (>5% factor activity).^[25]^ Hematuria is a frequent manifestation of hemophilia. Beck and Evans^[26]^ showed that 66% of hemophilia patient had a history of hematuria. Chakarova et al^[27]^ reported that during a 5-year follow-up of patients with hemophilia hematuria was found in approximately 25% of patients. Hematuria in hemophiliacs usually responds to conservative therapies and self-limited, some cases of severe hematuria have been reported which require cystectomy.^[28]^ Although the etiology of hematuria in hemophilia disease is often uncertain, it could be related to an underlying coagulation deficiency. In our case, there were no predisposing factors that would cause bleeding other than Hemophilia A, and the patient responded well to bypassing agents, suggesting that AGL was potentially associated with hemophilia A. Although spontaneous intramural hemorrhage of the ureter in hemophilia patients is a rare condition and has been stated in only 2 earlier cases not defined as AGL,^[7,29]^ no AGL was reported in hemophilia patients.

Until 2008, most patients were diagnosed with AGL after nephrectomy. First, Cardin et al^[10]^ reported a conservative approach case with subepithelial hematoma. In a case, because hematuria could not be managed conservatively, selective arterial embolization was applied.^[11]^ Recurrent cases of AGL have not been observed in patients managed conservatively in the literature. APCC and rFVIIa remain the mainstays for the treatment of bleeding in hemophilia patients with inhibitors. In our case, 4500 IU of FEIBA intravenously twice daily for 5 days was begun. The patient's macroscopic hematuria responded within 5 days and the frequency of FEIBA reduced once a day. During the follow-up period, abdominal CT at 3 months showed that the lesion had disappeared. We did not give long-term FEIBA prophylaxis and no bleeding episode was observed at a 1-year follow up. However, a single case may not be sufficient for long-term treatment plan. As the reported cases increase in the literature, treatment strategies may change. In the patient who has an underlying history of coagulopathy, nephrectomy can be avoided when there is awareness of AGL.

To our knowledge, this is the first case report of AGL in a patient with hemophilia A. Although rarely seen, AGL should be considered in the differential diagnosis of renal pelvic hemorrhage.

## Author contributions

**Conceptualization:** Taha Koray Sahin, Gulay Sain Guven, Ibrahim Celalettin Haznedaroglu.

**Data curation:** Taha Koray Sahin, Elifcan Aladag, Emil Settarzade.

**Formal analysis:** Taha Koray Sahin, Emil Setterzade, Elifcan Aladag, Salih Aksu.

**Methodology:** Taha Koray Sahin, Emil Settarzade.

**Supervision:** Gulay Sain Guven, Ibrahim Celalettin Haznedaroglu, Salih Aksu.

**Writing – original draft:** Taha Koray Sahin.

**Writing – review & editing:** Gulay Sain Guven, Ibrahim Celalettin Haznedaroglu.

## References

[1] Antopol W Goldman L Subepithelial hemorrhage of renal pelvis simulating neoplasm. Urol Cutan Rev 1948;52:189–95. 18859849

[2] Eccher A Brunelli M Gobbo S Subepithelial pelvic hematoma (Antopol-Goldman lesion) simulating renal neoplasm: report of a case and review of the literature. Int J Surg Pathol 2009;17:264–7. 19164409 10.1177/1066896908330482

[3] Iczkowski KA Sweat SD Bostwick DG Subepithelial pelvic hematoma of the kidney clinically mimicking cancer: report of six cases and review of the literature. Urology 1999;53:276–9. 9933039 10.1016/s0090-4295(98)00507-x

[4] Altay B Barişik CC Erkurt B Subepithelialpelvichematoma of the kidney (Antopol-Goldman Lesion). Turk J Urol 2015;41:48–50. 26328199 10.5152/tud.2014.48208 PMC4548647

[5] Şeker KG Şam E ⊠zdemir O A rare case mimicking collecting system tumor: antopol-goldman lesion. Bull Urooncol 2018;17:150–2.

[6] Jeevanandham B Dhanapal V Ramachandran R Induction chemotherapy causing pseudolesion in the kidney-antopol-goldman lesion: a radiological diagnosis and follow up with review of literature international. J Scientific Res 2018;7:6–7.

[7] Ameri A Martin R Vega R Successful management ofintramural ureteral hemorrhage in a patient with factor VIII deficiencyand high-titer inhibitor. J Thromb Haemost 2004;2:2273. 15613051 10.1111/j.1538-7836.2004.01041.x

[8] Ehrenforth S Kreuz W Scharrer I Incidence of development of factor VIII and factor IX inhibitors in haemophi- liacs. Lancet 1992;339:594–8. 1347102 10.1016/0140-6736(92)90874-3

[9] Chan IH Lam WW Wong KK Renal pelvis haematoma causing pelviureteric obstruction: a first case of Antopol-Goldman lesion in a neonate. J Paediatr Child Health 2010;46:361–2. 20642650 10.1111/j.1440-1754.2010.01783.x

[10] Cardin AL Marshall J Bhatt S Antopol-Goldman lesion of the kidney diagnosed by radiology: a case report of observation. Acta Radiol 2008;49:715–7. 18568566 10.1080/02841850802056009

[11] Branchereau J Leaute F Luyckx F Goldman Antopol syndrome associated with bilateral congenital severe factor V deficiency. Prog Urol 2010;20:604–7. 20832041 10.1016/j.purol.2009.12.002

[12] Labay GR Orkin LA Subepithelial hemorrhage of renal pelvis simulating neoplasm (Antopol-Goldman lesion). Mt Sinai J Med 1972;39:178–87. 4536797

[13] Viamonte M Roen SA Viamonte M Jr Subepithelial hemorrhage of renal pelvis simulating neoplasm (Antopol-Goldman lesion). Urology 1980;16:647–9. 7445318 10.1016/0090-4295(80)90581-6

[14] Levitt S Waisman J de Kernion J Subepithelial hematoma of the renal pelvis: a case report and review of the literature. J Urol 1984;131:939–41. 6708230 10.1016/s0022-5347(17)50720-x

[15] Oza KN Rezvan M Moser R Subepithelial hematoma of the renal pelvis (Antopol-Goldman lesion). J Urol 1996;155:1032–3. 8583554

[16] Kim SJ Ahn HS Chung DY Subepithelial hematoma of the renal pelvis simulating neoplasm (Antopol-Goldman lesion). Urol Int 1997;59:260–2. 9444747 10.1159/000283076

[17] Demirkan NC Tuncay L Düzcan E Subepithelialhaematoma of the renal pelvis (Antopol-Goldman lesion). Histopathology 1999;35:282–3. 10469224 10.1046/j.1365-2559.1999.0781d.x

[18] Villar Pastor CM López Beltrán A Alvarez Kindelán J Subepithelial hemorrhage of renal pelvis (Antopol-Goldman lesion). Report of 4 cases and review of the literature. Actas Urol Esp 2000;24:805–9. 11199297 10.1016/s0210-4806(00)72551-4

[19] Rodríguez Alonso A González Blanco A Cespón Outeda E Subepithelial hematoma of kidney pelvis and ureter: Antopol-Goldman lesion. Actas Urol Esp 2002;26:133–5. 11989427 10.1016/s0210-4806(02)72746-0

[20] Duffau P Morel D Basseau F Antopol-Goldman lesion: a rare cause of hematuria. Nephrol Ther 2005;1:131–4. 16895677 10.1016/j.nephro.2004.11.001

[21] Ortiz Gorraiz M Campaña Gutiérrez MA Subepithelial hematoma of the renal pelvis producing a filling defect in the upper urinary tract: radiological study. Arch Esp Urol 2005;58:354–9. 15989101 10.4321/s0004-06142005000400012

[22] Yapanoglu T Kocaturk H Aksoy Y Subepithelial hematoma of the renal pelvis (Antopol-Goldman lesion): a case report. Turkish J Urol 2009;35:159–61.

[23] Sánchez Zalabardo D De Pablo Cárdenas A Fuertes Zárate A Antopol-Goldman lesion: a rare clinical entity in the differential diagnosis of macroscopic hematuria. Arch Esp Urol 2012;65:258–62. 22414456

[24] Aguilar-García JJ Vargas-Serrano B Subepithelial pelvic hematoma. Actas Urol Esp 2012;36:620–3. 22999346 10.1016/j.acuro.2012.07.003

[25] White GC Rosendaal F Aledort LM Definitions in hemophilia—recommendation of the scientific subcommittee on factor VIII and factor IX of the scientific and standardization committee of the international society on thrombosis and haemostasis. Thromb Haemost 2001;85:560. 11307831

[26] Beck P Evans KT Renalabnormalities in patients with haemophilia and Christmas disease. Clin Radiol 1972;23:349–54. 5046533 10.1016/s0009-9260(72)80064-3

[27] Chakarova P Šukarova E Chakarov R Renalchanges in haemophilia A. Trakia J Sci 2005;3:52–5.

[28] Washino S Hirai M Kobayashi Y Heavy hematuri are quiring cystectomy in a patient with hemophilia A: a case report and literatüre review. BMC Urol 2015;15:84. 26268821 10.1186/s12894-015-0076-8 PMC4535382

[29] Kirbas I Ultrasound and computed tomography findings of spontaneous intramural hemorrhage of renal pelvis and ureter in patient with hemophilia A. Urology 2008;72:1030–2. 18701152 10.1016/j.urology.2008.06.012

